# Protein profile of the Escherichia coli strain, BW25113, exposed to two novel iron-halide compounds: Fe(Hampy)2Cl4 and Fe(Hampy)2Br4

**DOI:** 10.1099/acmi.0.000783.v4

**Published:** 2025-01-28

**Authors:** Nusrat Abedin, Sarah Wagner, Yukta Sanjay Khalkar, Zulekha Johnson, Biola F. Egbowon, Alan J. Hargreaves, Anthony J. Fitzpatrick, Amanda K. Miles, Felix Dafhnis-Calas

**Affiliations:** 1 School of Life Sciences, Faculty of Medicine and Health Science, University of Nottingham, Nottingham, NG7 2RD, UK; 2 Institute of Food Science and Technology (IFST), Bangladesh Council of Scientific and Industrial Research (BCSIR), Dr Qudrat-I-Khuda Road, Dhanmondi, Dhaka 1205, Bangladesh; 3 John van Geest Cancer Research Centre, Nottingham Trent University, Nottingham NG11 8NS, UK; 4 School of Science and Technology, Nottingham Trent University, Clifton Lane, Nottingham NG11 8NS, UK

**Keywords:** antimicrobial compound, antibiotic resistance, *Escherichia coli*, pathogens, proteomics

## Abstract

The mortality rate and economic burden of infections caused by antimicrobial-resistant pathogens are increasingly higher. This frustrating scenario emphasizes the urgent need for developing new antimicrobial drugs. We have previously addressed this problem by studying the antimicrobial activity of two novel iron-halide complexes, Fe(Hampy)_2_Cl_4_ (iron tetrachloride) and Fe(Hampy)_2_Br_4_ (iron tetrabromide). Both compounds showed bactericidal and antibiofilm activities against bacteria with an antimicrobial resistance phenotype. Herein, we used a proteomic approach to investigate the proteomic profile of bacterial cells previously exposed to both iron-halide complexes. For this study, the *Escherichia coli* strain, BW25113, was used as a model to facilitate the rapid identification of deregulated proteins. Heat map analysis of the common deregulated proteins highlighted that both complexes caused the downregulation of proteins associated with key metabolic pathways, biofilm formation, cell envelope biogenesis and iron ion binding. In addition, a network study suggested that the most influential proteins of the tetrachloride activity were those involved in the TCA cycle, oxidative phosphorylation, iron ion homeostasis and carbon/secondary metabolism. This protein–protein interaction analysis also hinted that the main drivers of the tetrabromide activity were proteins involved in translation, ribosomal biogenesis and cell motility. The above results strongly suggested how the presence of different halide ligands could be used to generate compounds with potentially different molecular mechanisms. Importantly, the findings of this study can also be used as a reference to compare with the protein profile of bacteria exposed to future variants of the iron-halide complexes.

## Graphical abstract

Characterizing the proteomic profile of the *Escherichia coli* strain, BW2513, exposed to two novel iron-halide complexes: iron tetrachloride and iron tetrabromide.

## Data Summary

Data generated during this study are provided in full within the published article and supplementary materials.

## Introduction

Antibiotics are one of the most important discoveries in therapeutic medicine. Their main mechanisms of action are based on targeting cell wall structure, bacterial metabolism or nucleic acid and protein synthesis. Unfortunately, the beginning of the antibiotic era had been associated with the emergence of antimicrobial-resistant bacteria. The latter together with the declining antibiotic pipeline has led to the rise of infectious diseases that are proving extremely difficult to treat [1,5]. This worrying scenario emphasizes the urgent need for developing new antimicrobial drugs that target distinct cellular functions [6,9]. Several research groups addressed this challenge by studying the antimicrobial activity of novel metal complexes formed by a well-defined arrangement of ligands around a metal centre [10,15]. These studies highlighted the diverse modes of action of the metal-based compounds as antimicrobial. The novel mechanism of action includes the targeting of the cell membrane, genetic material and the cellular pleiotropic effects on microbial cells mediated by the rise of reactive oxygen species (ROS).

A critical aspect in the discovery of novel antibacterial agents is the understanding of their mechanism of action [16]. Proteomic and transcriptomic analyses represent novel tools to unravel the mechanism of antibacterial agents [17,19]. These global technologies have the ability to highlight the characteristic signatures of a specific stress type and therefore reflect the unique response of the cell to the inhibition or activation of certain biological functions. The effectiveness of this approach was shown by Wang *et al.* in 2023. The authors used comparative proteomics to investigate the bacteriostatic mechanism of Ga(III) against *Pseudomonas aeruginosa*. The experimental findings of this study revealed the downregulation of metabolic pathways including glycolysis, TCA cycle, aa metabolism and a decrease in protein and nucleic acid synthesis. Moreover, a reduced level of proteins linked to the pathogenesis of *P. aeruginosa* was identified [20].

**Fig. 1.**
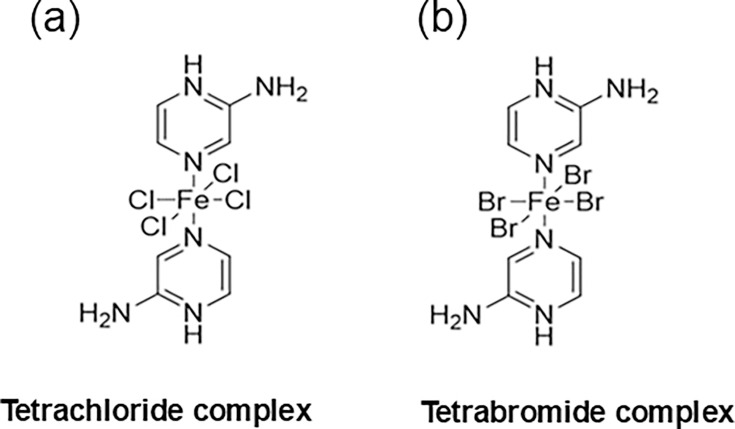
Chemical structure of the iron-halide complexes. (**a**) Iron tetrachloride complex, Fe(Hampy)_2_Cl_4_, 389.87 g mol^−1^. (b) Iron tetrabromide complex, Fe(Hampy)_2_Br_4_, 567.69 g mol^−1^.

In the previous work, we explored the antibacterial activity of two novel iron-halide complexes, Fe(Hampy)_2_Cl_4_ and Fe(Hampy)_2_Br_4_ (Fig. 1). Both complexes were synthesized through redox chemistry and then analysed by using X-ray crystallography. These compounds have an iron atom ligated to two aminopyrazinium molecules (Hampy) in the axial positions. The tetrachloride complex holds four chloride ligands in the equatorial position in contrast to the four equatorial bromides present in the tetrabromide complex. Our study demonstrated that both iron-halide complexes showed bactericidal and antibiofilm activities against bacteria with an antimicrobial resistance phenotype [21]. The antimicrobial activity was tested against three different bacterial species: (i) the methicillin-resistant *Staphylococcus aureus* NCTC 12493, (ii) the *P. aeruginosa* strain PAO1 and (iii) the *Escherichia coli* NCTC 10418.

Herein, we used a proteomic approach to investigate the effect of both iron-halide complexes on the proteomic profile of a bacterial cell. To achieve this, the *E. coli* strain *BW2513* was first exposed to the two iron-halide complexes. Our reason for choosing this strain was that the proteomic database of the *BW2513* bacteria strain is well characterized, which allows rapid identification of peptides via MS (Swiss-Prot *E. coli* K-12 BW25113 database, July 2018). Cell lysates from the bacteria were first analysed by MS in both sequential window acquisition of all theoretical mass spectra (SWATH) and information-dependent acquisition (IDA) modes to determine fold change differences due to the effects of the two iron-halide complexes. Gene Ontology (GO) analysis and protein–protein network interaction studies of the significantly deregulated proteins identified in the MS study were then carried out. These analyses led to the identification of the molecular target areas and the most influential proteins associated with the mechanism of action of the tetrachloride and tetrabromide iron complexes.

## Methods

### Bacterial cell growth

Frozen glycerol stocks of the *E. coli* BW25113 were grown on Luria–Bertani (LB) agar for 16 h, at 37 °C. Single colonies of the *E. coli* BW25113 were then pre-inoculated in 5 ml of LB broth followed by a 16-h incubation at 37 °C. Next, 500 µl of the 16-h culture was inoculated into a fresh 50 ml LB broth containing the tetrachloride and tetrabromide compounds at 1 and 0.5 mM concentrations, respectively. These sub-growth inhibitory concentrations of the iron-halide complexes were defined in a previous experiment by using a Miles and Misra method [22]. In addition, a 50 ml LB broth was cultured without exposure to the iron-halide complexes. The starting concentrations of the treated and untreated bacteria cultures were 4–5×10^5^ c.f.u. ml^−1^. Both treated and untreated cultures were incubated at 37 °C for 16 h with rotatory aeration at 180 r.p.m. The study involved three replicates per treatment group including (a) no_iron complex, (b) iron_tetrachloride and (c) iron_tetrabromide.

### Protein extraction, clean-up and trypsinization

Bacterial cells were first harvested by centrifugation of the 50 ml culture at 600 ***g*** for 15 min. Proteins were then extracted by homogenization of the cell pellet in 100 µl cell lysis solution [9.5 M urea (Melford, UK)/2% (v/v) DTT (Melford)/1% (v/v) *N*-octyl-beta-glucopyranoside (Apollo Scientific Limited, UK) containing 1% (v/v) protease inhibitor cocktail (Sigma, UK)]. The cell lysate was further solubilized using three rounds of sonication of 5 min each with 5-min pause on ice between cycles. The input parameters of the sonicator were 120 V and 40/60 Hz. Next, the debris was removed by centrifugation at 6700 ***g*** for 15 min at 4 °C. One in ten dilutions of the supernatant was used to determine the protein concentration by using the Pierce BCA Protein Assay Kit (Thermo, UK) prior to storage at −80 °C. Following quantification, each sample was normalized to a total mass of 100 µg by dilution in 50 mM tri-ethyl ammonium bicarbonate before reduction (5 mM DTT at 56 °C for 20 min) and alkylation (15 mM iodoacetamide at room temperature for 15 min). All samples were gently vortex mixed and incubated overnight for 18 h at 37 °C with MS grade trypsin (Promega, UK) at a 20:1 protein/protease ratio (w/w). Post-trypsinization, 0.5% (v/v) formic acid was added to reduce the pH and halt trypsin activity. All samples were then dried down at 60 °C and stored at −80 °C before resuspending in liquid chromatography MS grade acetonitrile [5% (v/v)] in 0.1% (v/v) formic acid for subsequent analysis.

### MS

Each sample was analysed on a Sciex TripleTOF 6600 mass spectrometer in both SWATH (to generate fold change data) and IDA modes. The mass spectrometer was coupled in line with an Eksigent 425 LC system running in micro flow (5 µl min^−1^) (mobile phase A, 0.1% formic acid; B, 100% acetonitrile+0.1% formic acid). In brief, 8 µg of each sample was injected onto a YMC-Triart C_18_ trap column (5 mm, 3 µm and 300 µm ID) at a flow rate of 10 µl min^−1^ in mobile phase A for 2 min. The sample was then eluted off the trap column and onto the YMC-Triart C_18_ analytical column (15 cm, 3 µm and 300 µm ID) that was in line with a Sciex TripleTOF 6600 Duospray Source using a 50 µm electrode in positive mode, +5500 V. The following linear gradients were used: for IDA, mobile phase B increasing from 3 to 30% over 68 min and 40% B at 72 min followed by column wash at 80% B and re-equilibration (87-min total run time). For SWATH, the following linear gradients were used: 3–30% B over 38 min and 40% B at 43 min followed by wash and re-equilibration as before (57-min total run time). IDA acquisition mode was used with the top 30 ion fragmentations. The time-of-flight mass spectrometry was acquired at m/z 400-1250 and the product ion scan was selected at m/z 100-1500. The accumulation time of the product ion was set to 50 ms, giving a cycle time of 1.8 s. The dynamic exclusion was used for 15 s with rolling collision energy. SWATH acquisition was performed using 100 variable windows followed a previous optimization on sample type. The time-of-flight mass spectrometry was acquired at m/z 400-1250 with a 25 ms accumulation time giving 2.6 s per cycle. IDA data were searched together using ProteinPilot 5.0.2 (iodoacetamide alkylation, thorough search with emphasis on biological modifications) against the Swiss-Prot *E. coli* K-12 BW25113 database (July 2018). SWATH data were analysed using Sciex OneOmics software [23] extracted against the locally generated library with the following parameters: 12 peptides per protein, 6 transitions per peptide, the extracted ion chromatogram width 30 ppm and 5-min retention time window.

### GO annotation and pathway analyses

Selected proteins with a confidence value of ≥50% [23] were submitted to the online tool: DAVID Functional Annotation Bioinformatics Microarray Analysis [24] (https://david.ncifcrf.gov/). Here, the uploaded proteins were subjected to GO and pathway analyses. The GO analysis associates the applied proteins to one or more categories, e.g. biological processes, cellular components and molecular functions, and ranks the associated subcategories based on frequency. Parallel to this, the filtered protein list was subjected to a pathway analysis using the Kyoto Encyclopedia of Genes and Genomes (KEGG) [25]. Here, pathways are ranked based on the coverage of the supplied proteins with proteins associated with specific pathways annotated in the software.

### Network inference analysis

To evaluate the interaction of proteins and for the definition of key drivers within the influenced biological conditions, artificial neural network (ANN) inference modelling was applied [26] using proteins with a confidence value of ≥70%. The generated interaction intensities can be negative or positive values and can range from small to large influences on the target protein. In this study, a three-layered multi-layer perceptron with backpropagation learning and sigmoidal activation function was used [27]. Monte Carlo Cross-validation was applied to prevent overfitting using a randomly assigned 60:20:20 split for training, testing and validation.

### Heat map

Selected proteins with a ≥50% confidence and the measured protein peak areas (normalized) were applied to MORPHEUS for the generation of a heat map (https://software.broadinstitute.org/morpheus/).

### Statistical analysis

The protein fold changes between the treated and the control were calculated to identify the intensity and directionality of induced protein changes. Statistical analysis was performed using GraphPad Prism version 8.0 (GraphPad Software, San Diego, CA) and Microsoft Excel. Data are expressed as mean±sem. Significant differences between the analysed groups were evaluated using unpaired Student’s t-test analysis, *P*≤0.05. Analysis of the data was only performed with deregulated proteins, showing a confidence cutoff of ≥50% [23].

### Chemical synthesis

Fe(Hampy)_2_Cl_4_ and Fe(Hampy)Br_4_ were synthesized according to the previous literature methods [21,28].

## Results

### The proteomic profile of commonly deregulated proteins highlights the downregulation of key biological processes in bacteria exposed to iron-halide complexes

Proteomics has become an effective tool to provide information on the potential antimicrobial mechanisms of action, as it allows the qualitative analysis of a large number of proteins simultaneously. Here, we have used a proteomic approach to investigate the proteomic profile of a bacterial strain previously exposed to the iron tetrabromide or the iron tetrachloride complex.

**Fig. 2.**
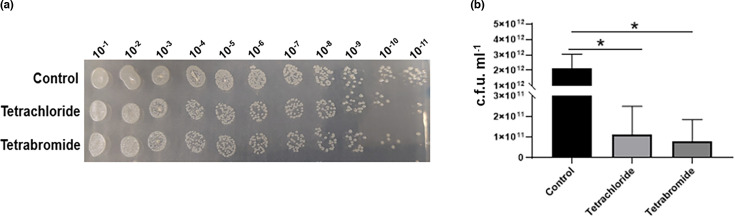
Growth of the *E. coli BGW2513* strain exposed to the sub-total growth inhibitory concentrations of the tetrachloride and tetrabromide compounds. The bacterial cells were grown at 37 °C in the presence of iron tetrabromide and iron tetrachloride, at 0.5 and 1 mM, respectively. (**a**) Miles and Misra experiment of the *E. coli* strain *BW2513* in the presence and absence of the iron-halide complexes. The image shows the results from the inoculation with 10^-1^–10^-11^ dilutions of the pure culture. (**b**) C.f.u. per millilitre of the treated and untreated bacteria. The differences between all growth values were analysed statistically by using the one-way ANOVA test and Turkey’s post hoc test, *P≤0.05. Error bars indicate the sem from three independent experiments.

The *E. coli BW2513* strain was used as a model since it has a very well-characterized protein database (Swiss-Prot *E. coli* K-12 BW25113 database, July 2018), which allows rapid peptide identification. Proteins were extracted from *E. coli* cells exposed to concentrations of the iron-halide complexes that caused a reduction in the growth rate but did not inhibit growth completely. It is known that such sub-inhibitory concentrations of an antibiotic can produce significant antibacterial effects [29,31]. Therefore, studying the proteomic changes of the cells treated with sub-inhibitory concentrations of the iron-halide compound could shed light on their antibiotic mechanism of action. The bacterial strain *BW2513* was first exposed to a range of concentrations of the iron-halide complexes, and then, a Miles and Misra experiment was carried out to identify sub-MIC, as defined by Kowalska-Krochmal and Dudek-Wicher in 2021 [32]. Fig. 2 highlights the results of the Miles and Misra experiment performed at the sub-inhibitory concentrations used in the proteomic study. These were chosen as concentrations that inhibited but did not completely prevent bacterial cell growth. The iron-halide complexes caused a decrease in c.f.u. over a 24-h period (Fig. 2a). There was a statistically significant difference between the growth rate of bacteria treated with the iron-halide complexes when compared to the control experiment (*P*≤0.05) (Fig. 2b).

**Fig. 3.**
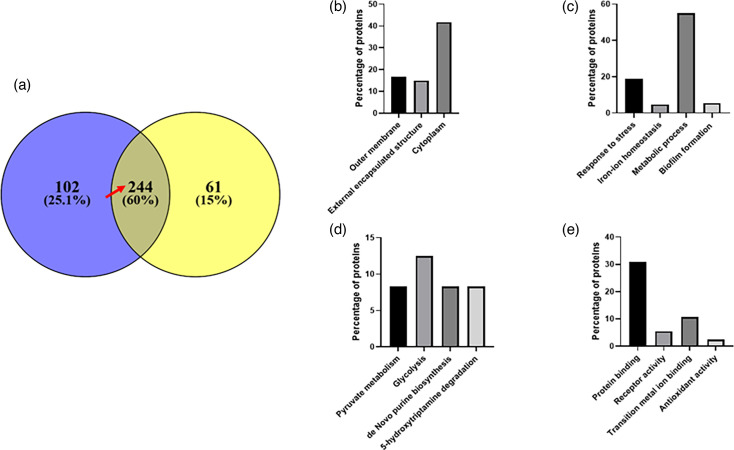
GO analysis of shared deregulated proteins from *E. coli* strain, BW25113, exposed to iron tetrachloride and iron tetrabromide complexes. (**a**) Venn diagram showing proteins that became deregulated upon exposure to each of the iron compounds. (**b**) Cell components, (**c**) biological processes, (**d**) KEGG pathways and (e) molecular function.

The proteomic analyses of cell lysates derived from treated and untreated *E. coli* resulted in the identification of 1077 proteins with a false discovery rate of 1%. Out of these 1077 proteins, 346 proteins from the iron tetrachloride and 305 proteins from the iron tetrabromide experiment were significantly (≥50% cut-off) deregulated compared to the untreated control. Among these proteins, 244 were shared by both treatments, whereas 102 and 61 protein changes were unique to the treatment with tetrachloride and tetrabromide, respectively (Fig. 3a).

To understand the underlying changes induced through the treatment, we carried out a GO annotation analysis [24] to gain insight into the cellular location and functional association of the shared proteins. Based on the GO annotation analysis, differentially expressed proteins were classified into four groups: (i) the biological process, (ii) the cell component, (iii) molecular functions and (iv) molecular pathways (Fig. 3). In the biological process category, proteins identified were those involved in response to stress (19%), iron ion homeostasis (4.8%), metabolic process (55%) and biofilm formation (5.4%). For the cellular component group, the proteins were part of the outer membrane (16.7%), external encapsulated structure (14.9%) or cytoplasm (41.7%). In the molecular function category, identified proteins were involved in protein binding (30.8%), receptor activity (5.4%), transition metal ion binding (10.7%) and antioxidant activity (2.4%). In addition, we also applied the 244 shared proteins to pathway analysis based on the KEGG database [25]. This highlighted an enrichment of pyruvate metabolism (8.3%), glycolysis (12.5%), *de novo* purine biosynthesis (8.3%) and 5-hydroxytryptamine degradation (8.3%).

**Fig. 4.**
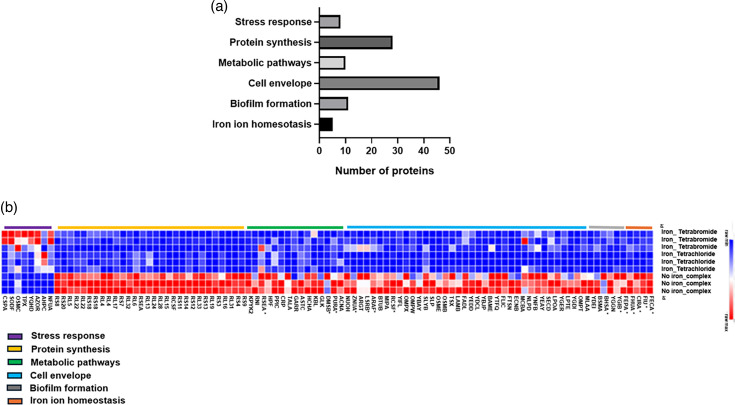
Protein profile of the *E. coli* strain, BW25113, exposed to the iron-halide complexes. (**a**) Enriched biological processes of the significantly changed proteins common to both iron-complex treatments. (**b**) Summary of the heat map view based on the shared deregulated proteins that contribute to the following GO terms: biofilm formation, stress response, protein synthesis, metabolic pathways and membrane proteins. Proteins highlighted with * were shared across multiple biological processes.

A heat map representation of the proteomic changes was generated with the common deregulated proteins that contribute to the following GO terms: biofilm formation, stress response, protein synthesis, metabolic pathways, iron ion homeostasis and membrane proteins (Fig. 4b) (*n*=96 proteins). These GO terms were chosen based on those previous reports, highlighting their role in antibiotic action and resistance [33,36]. The analysis revealed significant differences in the proteomic profile between the treated and untreated bacteria. Overall, 88 out of 96 commonly regulated proteins were downregulated in the treated cells in comparison with the control experiment. Interestingly, the shared upregulated proteins corresponded to the response to the stress category. Details of the specific identified proteins and their observed levels are indicated in Table S1 (available in the online version of this article).

### Identifying the differences in the protein profile of the bacteria treated with the iron tetrachloride and iron tetrabromide complexes

**Fig. 5.**
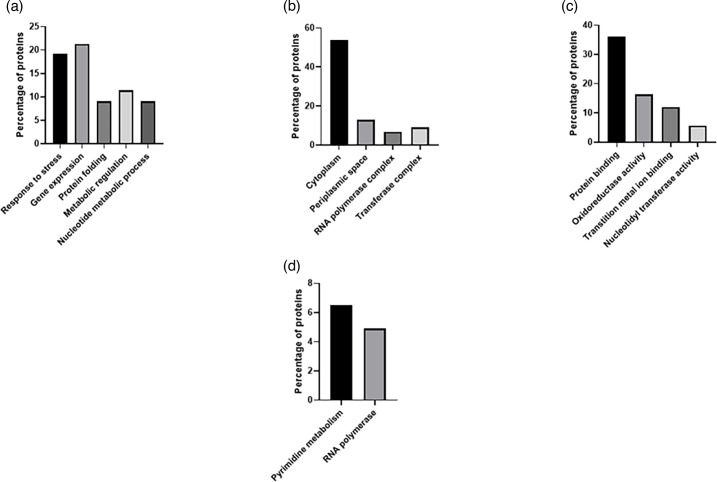
GO analysis of significant deregulated proteins from *E. coli* strain, BW25113, exposed to iron tetrabromide complexes. (**a**) Biological processes, (**b**) cell components, (**c**) molecular function and (d) KEGG pathways.

As previously mentioned, 61 out of 306 proteins and 102 out of 346 proteins were uniquely regulated by iron tetrabromide and iron tetrachloride, respectively (Fig. 3a). The GO annotation analysis of the differentially expressed proteins related to the tetrabromide experiment highlighted that in the biological process category, most proteins were involved in response to stress (19.2%) and gene expression (21.3%) (Fig. 5). Similarly, in the cellular component group, 54% of proteins were assigned to the cytoplasmic group, while 13% formed part of the periplasmic space. Also, a large proportion of the proteins assigned to the molecular function category were involved in protein binding (36%), while 16.3% were related to oxidoreductase activity. The KEGG pathway analysis highlighted an enrichment of the pyrimidine and RNA polymerase pathways with 6.5 and 4.9% of proteins, respectively.

**Fig. 6.**
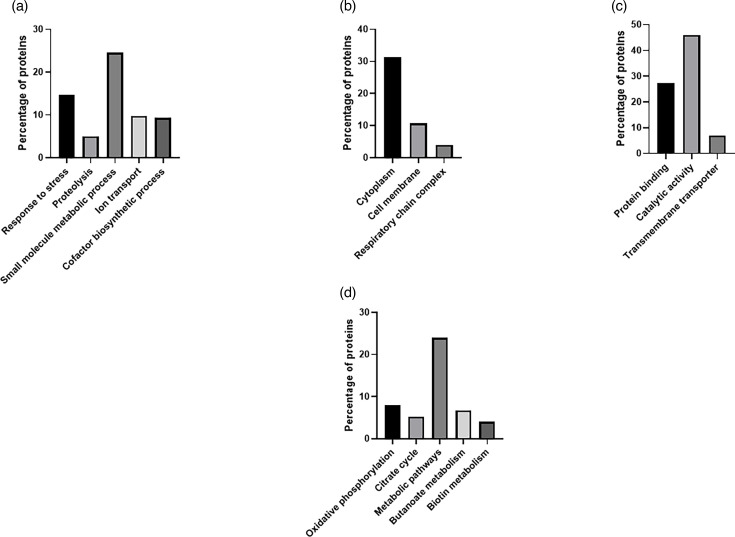
GO analysis of significant deregulated proteins from *E. coli* strain, BW25113, exposed to iron tetrachloride complex. (**a**) Biological processes, (**b**) cell components, (**c**) molecular function and (d) KEGG pathways.

Furthermore, as shown in Fig. 6, the enrichment analysis of the tetrachloride experiment revealed that in the biological process category, most proteins were involved in response to stress (14.7%), small-molecule metabolic process (24.5%), proteolysis (5%) and ion transport (9.8%). Moreover, in the cellular component group, 31.3% corresponded to the cytoplasmic part, while 10.7% were identified as envelope proteins. In the molecular function category, most of the identified proteins were involved in protein binding (27.4%), while 46% were related to a catalytic function. The KEGG pathway analysis revealed enrichment of the metabolic pathways (24%).

**Fig. 7.**
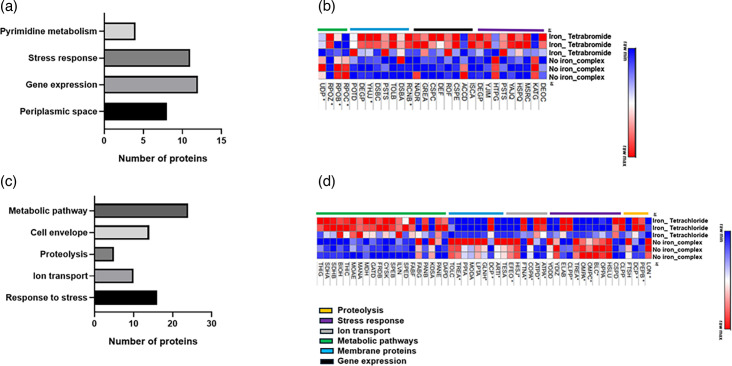
Differential proteomic profile of the *E. coli* strain, BW25113, exposed to the iron-halide complexes. (**a**) Enriched biological processes based on unique proteins specifically regulated in the presence of the tetrabromide compound. (**b**) Heat map view based on the proteins significantly deregulated by the tetrabromide complex. (**c**) Enriched biological processes based on unique proteins specifically deregulated in the presence of the tetrachloride compound. (**d**) Heat map view based on the proteins significantly deregulated by the tetrachloride complex. Proteins highlighted with * were shared across multiple biological processes.

Details of the unique tetrabromide-deregulated proteins and their relative levels are shown in Table S2. Overall, 64% of the deregulated proteins were upregulated in the treated cells in comparison with the control experiment. The proteins specifically upregulated by the tetrabromide treatment were associated with response to stress, cell envelope and gene expression. A heat map representation of these unique tetrabromide-regulated proteins is shown in Fig. 7b.

Similarly, the details of the unique tetrachloride-deregulated proteins and their relative levels are shown in Table S3. This table shows that 76% of those deregulated proteins were upregulated in the treated cells. A heat map representation of the expression profile of the unique tetrachloride-regulated proteins is shown in Fig. 7d. This study was generated with the proteomic data of deregulated proteins that contribute to the following GO terms: response to stress, metabolism, proteolysis, ion transport and cell envelope. Altogether, the results from this heat map indicate the upregulation of the proteins associated with proteolysis and metabolic processes. The analysis also highlights the downregulation of the proteins linked to cell membrane and stress response.

Overall, the GO and heat map analyses of those unique sets of deregulated proteins revealed an enrichment of proteolytic and ion transport activities in the tetrachloride but not the tetrabromide treatment. Among the proteolytic proteins, it is worth noting the downregulation of the Lon protease and the ATP-dependent Zn metalloprotease FtsH. Earlier reports had demonstrated the role that both proteases play in antibiotic resistance [37,39]. Conversely, the GO study highlighted the enrichment of proteins associated with gene expression functions in the tetrabromide but not in the tetrachloride-treated cells. In this regard, the tetrabromide treatment induced the downregulation of the AccD protein, the beta subunit of the acetyl-CoA carboxylase complex. The acetyl-CoA carboxylase catalyses the first step in fatty acid synthesis. The full name of the AccD protein is acetyl-coenzyme A carboxylase carboxyl transferase subunit beta [40]. In addition to its catalytic role, the AccD protein regulates the translation of itself and the alpha subunit of the acetyl-coenzyme A carboxylase [40]. Earlier work had demonstrated the role that this enzyme plays on antimicrobial resistance since inhibitors of its activity showed high antibiotic efficacy [41].

### Network analysis of significantly deregulated proteins highlights key molecular drivers of the *E. coli* response to the iron-halide complexes

The ANN algorithm was used for the identification of major drivers within the iron-halide-treated system. It is a stepwise algorithm that interrogates the proteomic data generated from the MS analysis. The algorithm iteratively quantifies the influence that multiple proteins might have on the relative level of a single protein, until all the identified proteins from the MS data are quantified this way. The entire interrogation process allows the discovery of the most influential proteins (central hubs) within the system while determining the intensity and the directionality of the protein–protein interactions. The association between protein pairs can be bi- or unidirectional as well as being either stimulatory or inhibitory [26].

**Fig. 8.**
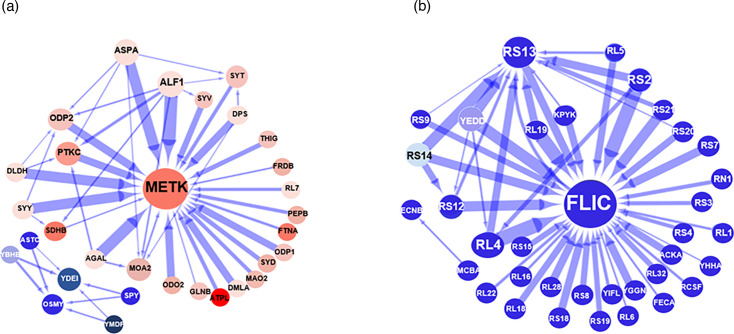
Interactome of the *E. coli* strain, BW25113, exposed to iron tetrachloride and iron tetrabromide complexes. Network analysis of the bacterial strain showing the 50 strongest interactions among the dataset of significant identified proteins. Red nodes are upregulated genes, and blue nodes are downregulated genes. The intensity of the colour is directly proportional to the degree of up- or downregulation. The blue arrow indicates inhibition. The thickness of the arrow is directly proportional to the strength of the interaction. (**a**) Iron tetrachloride network analysis. (**b**) Iron tetrabromide network analysis.

This *in silico* study allowed the determination of the main protein interactions that influence the response of the bacteria to each of the iron-halide compounds. For this, we applied an increasingly stringent selection approach to the protein list and only included proteins with a confidence value of ≥70% [23]. The proteins fulfilling this criterion were then subjected to the network inference analysis, in which we focussed on the top 50 strongest interactions across the analysed protein selection. Results from this network analysis revealed that the largest hub, and therefore one of the strongest influential drivers of the tetrachloride systems, was the *S*-adenosylmethionine synthase protein, MetK (Fig. 8a). This enzyme catalyses the formation of *S*-adenosylmethionine (AdoMet) from methionine and ATP. AdoMet is an essential metabolite required in an extensive range of biochemical reactions involving the transfer of methyl and adenosyl groups [42]. This network inference study also uncovered the directionality and intensity of MetK protein interactors within its network. All the components of this network were upregulated and exerted an inhibitory effect on the main hub protein, MetK. Enrichment analysis of the proteins that form part of the MetK interactome revealed that they are largely involved in catalysis, iron binding and transporting activities. Table S5 summarizes the proteins involved in each of the pathways highlighted.

Furthermore, an independent network within the top 50 interactions was also identified in the tetrachloride analysis (Fig. 8a). This interactome is composed of six downregulated proteins, and the nature of all the interactions was inhibitory. The two major hubs of this network were the osmotically inducible protein Y precursor (OsmY) and the uncharacterized protein YdeI (YdeI). OsmY is associated with chaperone-mediated protein folding and the hyperosmotic response [43]. The YdeI proteins are involved in the cellular response to hydrogen peroxide and single-species biofilm formation [44]. All the components of this network were downregulated and exerted an inhibitory effect on the main hub proteins, OsmY and YdeI.

The protein–protein interaction shown in the tetrabromide study revealed a different scenario (Fig. 8b). Firstly, the largest hubs and therefore the most influential drivers of the iron tetrabromide systems were the flagellin protein FliC and the 30S ribosomal protein, RS13. FliC is a subunit protein that polymerizes to form the filaments of bacterial flagella, while RS13 is a component of the 30S *E. coli* ribosome [45,46]. The other aspect that distinguished the iron tetrabromide interactome from the iron tetrachloride interactome was that all proteins in the iron tetrabromide interactome were downregulated. All the downregulated proteins in this tetrabromide interactome also exerted an inhibitory effect on the main drivers (RS13 and FliC hubs). Interestingly, more than 90% of the RS13 and FliC interactions were with proteins involved in translation and ribosome biogenesis. Further details of the functions associated with the main components of this tetrabromide interactome are shown in Table S4.

**Fig. 9.**
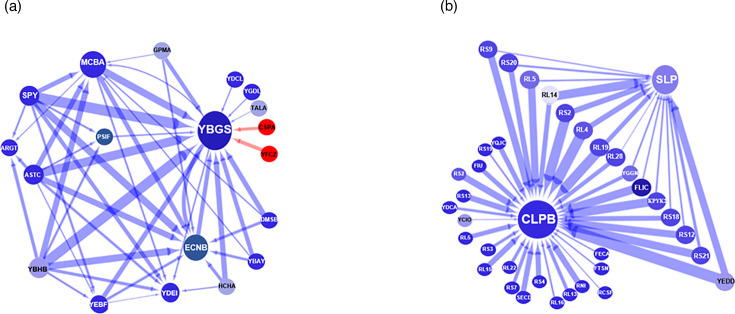
Interactome of the *E. coli* strain, BW25113, exposed to the iron tetrachloride and iron tetrabromide compounds. Network analysis of the biofilm strains shows the 50 strongest interactions among the shared proteins. Red nodes are upregulated genes, and blue nodes are downregulated genes. The intensity of the colour is directly proportional to the degree of up- or downregulation. Red arrows indicate stimulatory signals, while blue arrows show inhibition. The thickness of the arrow is directly proportional to the strength of the interaction. (**a**) Iron tetrachloride study. (**b**) Iron tetrabromide study.

We also implemented an additional network analysis based on those proteins commonly deregulated by both iron-complex treatments to see if the generated networks could also highlight differences in the key molecular drivers associated with each of the iron-halide complex (Fig. 9). This analysis showed that one of the more influential drivers of the iron tetrachloride system was the homeobox protein, YbgS, a putative homeobox protein with a DNA binding function [47]. Ninety per cent of the proteins in this network are downregulated and had an inhibitory effect on the level of the YbgS protein (Fig. 9a). Also, an enrichment study of the YbgS interactome indicated that they are mainly involved in metabolism, transportation and DNA regulatory activities. The only members of this network that were upregulated, but still have a downregulatory effect on YbgS, were the cold shock protein, CspA, and the UPF0381 protein, YfzZ (Fig. 9a). CspA is a stress response protein with DNA binding functions, while YfcZ is a protein of unknown function [48]. Additional details of the proteins that form part of the identified YbgS network are shown in Table S7.

The network inference based on shared deregulated proteins showed a different set of key molecular drivers for the iron tetrabromide experiment (Fig. 9b). The largest hub and more influential driver were the chaperone protein ClpB, which is part of the stress-induced system, involved in the recovery of the cell from heat-induced damage [49,50]. All the proteins from the ClpB cluster were downregulated and had a negative effect on the level of the chaperone protein ClpB. A further GO analysis showed a significant enrichment of ribosomal proteins (>50%) within this ClpB cluster. Similarly, the network associated with the tetrabromide compound identified the outer membrane protein SLP [51], as a second major hub. Thirty per cent of the proteins from the ClpB cluster interact with the proteins from the SLP hub. All the proteins from the ClpB/SLP cluster did have an inhibitory effect on the level of SLP. Further details of the main components of this ClpB cluster are shown in Table S6.

**Fig. 10.**
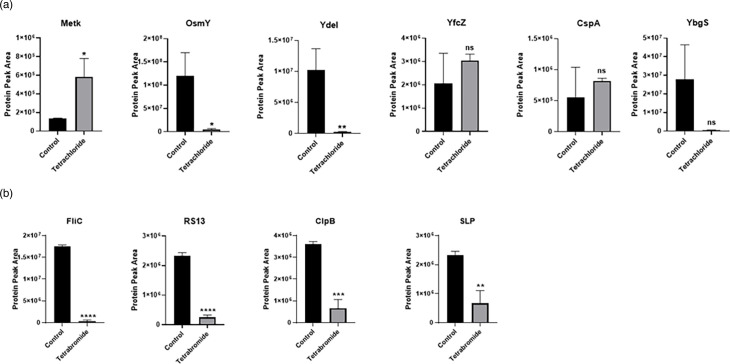
Comparative levels of the hub proteins in iron-halide treated and untreated cells. (**a**) Protein levels of the MetK, OsmY, YdeI, YfcZ, CspA and YbgS in untreated and treated bacterial cells exposed to the iron tetrachloride complex. (**b**) Levels of the FliC, RS13, ClpB and SLP in untreated and treated bacterial cells exposed to the iron tetrabromide complex. The differences between all values were analysed statistically by using unpaired Student’s t-test analysis, *P*≤0.05. Error bars indicate the sem; *n*=3.

Figure 10 shows the measured protein peak areas (obtained from the MS data) of proteins of interest based on their centrality on the generated networks in their natural and treated conditions. The proteins selected for this were identified as key drivers common to both iron tetrachloride and iron tetrabromide experiments. Significant differences were observed in three out of six of the key regulators associated with the iron tetrachloride treatment including MetK, OsmY and the YdeI proteins. However, there were no statistically significant differences in the observed levels of the CspA, YfcZ and YbgS proteins (Fig. 10a). Interestingly, all the main drivers of the iron tetrabromide study showed a statistically significant reduction in their protein level intensities following treatment with the iron-halide complex (Fig. 10b).

## Discussion

Antimicrobial resistance represents one of the biggest threats to global health. Scientists are trying to overcome this problem by developing antibiotics with novel mechanisms of action [1,4]. We sought to address this challenge by studying the antimicrobial activities of two novel iron-halide complexes, tetrabromide [Fe(Hampy)_2_Br_4_] and tetrachloride [Fe(Hampy)_2_Cl_4_] [21]. In the current study, we performed a proteomic analysis to investigate the proteomic profile of the bacterial cells previously exposed to the tetrabromide and tetrachloride complexes. The heat map visualization of the shared deregulated proteins showed a downregulated protein profile in the treated cells when compared to the control experiment. Further interrogation into the proteins that were downregulated revealed that exposure of the bacteria to the iron complexes decreased the levels of cell envelope proteins as well as downregulating those proteins involved in biofilm formation, metabolic pathways and iron ion binding. This downregulatory trend is important because it involves some of the biological processes that play a role in antibiotic action and resistance [33,36].

Moreover, the heat map study showed a rise in the levels of various stress response proteins upon exposure to the iron compounds. Interestingly, most of the identified upregulated proteins were involved in the oxidative stress response [52,56], namely, (i) the Fe/S biogenesis protein NfuA, involved in iron–sulphur cluster biogenesis under severe oxidative stress [57,58]; (ii) the FMN-dependent NADH: quinone oxidoreductase, AzoR, involved in resistance to thiol-specific stress [59]; (iii) the thiol-specific peroxidase, AhpC, which catalyses the reduction of hydrogen peroxide and organic hydroperoxides to water and alcohol, respectively [60,61]; (iv) the NADP-dependent alcohol dehydrogenase enzyme, YqhD, which protects from the harmful effect of lipid peroxidation-derived aldehydes [54,62]; (v) the thiol-specific peroxidase, TpX, which catalyses the reduction of hydrogen peroxide and organic hydroperoxides to water and alcohols, respectively [55,63]; and (vi) the superoxide dismutase (Fe-Zn), SODF, which plays a key role in the removal of superoxide radicals [64].

The above findings are in line with earlier studies reporting how the antibacterial activity of metal-based nanoparticles, including their halogenated derivatives, was associated with oxidative stress [65,66]. The antimicrobial mechanisms of these metal-based nanoparticles have been associated with destabilization of the bacterial cell wall and membrane, followed by changes in its permeability, induction of toxicity and oxidative stress by the generation of ROS. For instance, Li *et al.* [66] obtained *N*,*N*,*N*-trimethylammonium bromide-gold nanoclusters (MUTAB-AuNCs) that exhibited an effective antimicrobial activity against Gram-positive and Gram-negative bacteria (*Streptococcus pneumoniae*, *P. aeruginosa* and *E. coli*) and fungi (*Candida albican*). The study detected an increase in the bacterial ROS levels by using an ROS assay kit based on the 2′,7′-dichlorofluorescein diacetate reagent [66]. These results are also consistent with a previous study, highlighting an association between a general downregulatory effect of some antimicrobial compounds and a compensatory stress response [67].

Furthermore, the GO study of the unshared significantly deregulated proteins highlighted the downregulation of antibiotic resistance-associated proteins. In this regard, the bacterial cells exposed to the iron_tetrachloride showed reduced levels of the Lon and FtsH proteases. The bacterial cells treated with iron_tetrabromide exhibited downregulation of the AccD protein. The roles that the above proteins play in antibiotic resistance have been demonstrated by various research groups [68,72]. Harms *et al.* [68] demonstrated that the Lon protease enhances the persister populations in *E. coli* by increasing their resistance to antibiotics [68]. The function that the AccD protein plays on antibiotic resistance was shown by Freiberg *et al.* [70].

More importantly, both the downregulation of the Lon- and FtsH-mediated proteolysis and the AccD metabolic function contribute to an overall reduction in cell survival [72,73]. It is known that the Lon protease acts as a global regulator of various biological processes, such as biofilm formation, proteolysis of damaged proteins and bacterial virulence [73]. Similarly, the ATP-dependent protease, FtsH, has been reported to be an essential protein in several bacteria, due to its role in maintaining membrane protein homeostasis [74]. The essential role of the acetyl-CoA carboxylase on cell survival is related to its requirement for the synthesis and maintenance of cellular membranes [75]. Therefore, the downregulation of these three proteins can contribute to the development of molecular mechanisms similar to the mode of action of metal complexes with antimicrobial activity [10,15].

We also used a machine learning-based network analysis tool to determine the most influential protein–protein interactions associated with the exposure of the bacteria to the iron-halide complexes. As we pointed out, this software interrogates the proteomic data by iteratively quantifying the influence that multiple proteins might have on the level of a single protein [26]. The network generated from the iron tetrabromide experiment showed the flagellar protein FliC as the central node interacting with several ribosomal proteins such as RS13, RL14, RL4, RS12 and RS2.

These supramolecular structures are directly involved in bacterial mobility, host colonization, evasion of the host immune system and biofilm formation [76,77]. Our findings agree with an *E. coli* proteomic study that identified FliC as one of the hub–bottlenecks associated with the stress response network [78]. Moreover, the central role of the FliC protein is in line with an earlier study, reporting the dominant role of the flagellar protein in bacterial virulence [79]. These authors demonstrated that the motility of the uro-pathogenic *E. coli* strain (UPEC) was hindered by the antimicrobial activity of cranberry compounds. They also established that the inhibition of motility was the result of a decreased level of the FliC protein. An earlier transcriptomic study carried out by the same group revealed the downregulation of the *fliC* gene upon exposure of the UPEC strain to the cranberry-derived proanthocyanidins [80]. These findings are also consistent with the crucial function of FliC in the pathogenesis of bacteria such as *Proteus mirabilis*, *Salmonella* species, *Helicobacter pylori* and entero-pathogenic *E. coli* [81,83]. Therefore, the downregulation of the FliC proteins has a negative effect on the mechanisms that not only promote antimicrobial resistance but also bacterial virulence and biofilm formation. The antibiofilm property of the cranberry juice has been associated with bacterial cell adhesion in the first stages of biofilm development [84].

The networks generated from the iron tetrabromide study also showed that all ribosomal proteins within this network were downregulated and had an inhibitory influence on FliC. The latter is consistent with some of the findings of Fan *et al.* in 2016 who suggested an association between the inhibition of flagella biosynthesis and fidelity of protein synthesis [85]. Overall, the downregulated state of the tetrabromide interactome observed in the current study agrees with reports highlighting how energy-costly processes such as flagellar biosynthesis and ribosome biogenesis are tightly regulated in bacterial stress response [86,87].

However, the interactome generated from the iron tetrachloride study showed a different scenario. Firstly, the study revealed an upregulated MetK protein as the most prominent hub. Secondly, the analysis showed that all the proteins from this MetK network were upregulated and exerted an inhibitory effect on the level of MetK. This negative effect on the level of the upregulated MetK network suggests that the system was trying to re-establish homeostasis upon exposure to the tetrachloride compound. The need for inhibiting MetK expression might be related to the fact that the chemical reactions that use S-Adenosyl methionine as a substrate also lead to the generation of inhibitory byproducts, such as *S*-adenosylhomocysteine (SAH), 5′-methylthioadenosine (MTA) and 5′-deoxyadenosine [88]. Increased levels of these byproducts cause the inhibition of cell growth and metabolism [89,90]. Therefore, the return to normal levels of MetK may reflect the urgency for decreasing the level of inhibitory byproducts. An alternative explanation might be related to the fact that in bacteria, SAH synthesis and the TCA cycle are known to compete for the availability of succinyl CoA [91].

GO analysis of the proteins included in the MetK cluster showed an enrichment of iron ion homeostasis processes, TCA cycle, oxidative phosphorylation and small-molecule metabolic pathways. The upregulated status of the MetK protein suggests that this enzyme plays a central role in the stress generated by the tetrachloride compound. Others have shown this association between the MetK protein and stress response [92]. The authors of that study stated that microbes under abiotic stress produce the methylation product betaine (*N*,*N*,*N*-trimethylglycine) as an osmo-protectant. Furthermore, the inclusion of the small-molecule metabolic pathway within the MetK cluster is in line with the positive effect that this enzyme plays in the biosynthesis of small-molecule metabolites [93]. This suggests that the observed increased level of SAM synthase may lead to an increase in the synthesis of the small-molecule metabolites. However, the tetrachloride network did not show any stimulatory effect from the MetK protein on any of the components of the MetK-hub. The network only revealed an inhibitory signal of MetK protein on a protein of unknown function, the molybdenum cofactor biosynthesis protein, MoaB [94].

In summary, the results of both network and heat map analyses suggested that iron tetrachloride and iron tetrabromide compounds caused a reduction in the levels of the metabolic, biofilm and envelope proteins by exploiting different functional networks. The observed dissimilarities highlighted important differences in the network of the more influential deregulated proteins associated with exposure to each of the iron-halide complexes. The differences between the networks associated with the iron-halide compounds are in line with the report from Mughal *et al.* in 2006. The authors discovered a rise in the antimicrobial activity of the flavonoids with the increasing electronegativity of the halide ligands [95]. In the same line, others have shown a correspondence between antimicrobial activity and the hydrophobic effect of the halide ligand [96].

As discussed earlier, two additional network inference analyses were performed based on the shared proteins commonly deregulated by both iron-halide complexes. We hypothesized that a network inference analysis based on a shared set of proteins should also reveal dissimilar networks. The results of this study showed the putative DNA binding protein, YbgS, as the main key driver of the iron tetrachloride network. The suggested association between DNA binding proteins with a compound that shows antimicrobial activity has been highlighted in previous studies. This includes a study highlighting the targeting of the DNA gyrase enzyme by known antibiotics such as norfloxacin and ciprofloxacin [97]. However, the protein-protein interaction study based on shared deregulated proteins revealed that the biggest driver of the tetrabromide network corresponded to the chaperone protein, ClpB. The central role of this protein has been demonstrated in a study in which the deletion of the gene decreased bacterial growth and stress tolerance in *E. coli* [98]. Therefore, the results from the network analyses of the shared deregulated proteins highlighted the differences in the proteomic profiles associated with exposure to both iron complexes.

We acknowledge that a limitation of this study is the lack of biological validation of the proteomic findings. Therefore, further experiments are needed to test the predictions made by using the different bioinformatic tools. This could, for example, involve the implementation of Western blot assays and enzyme activity assays, among others, to test the hypotheses derived from the proteomic study.

## Conclusion

Taken together, our data suggest that the molecular effect of both iron-halide complexes involves the downregulation of proteins associated with biofilm formation, key metabolic pathways, cell envelope biogenesis and iron ion homeostasis. The proteomic data also implied that this downregulatory effect could be associated with an increase in the level of ROS, as the cells exposed to both compounds showed the upregulation of oxidative stress responses. These molecular changes concur with the mode of action reported for other metal complexes and halogenated agents with antimicrobial activity [10,15]. Therefore, the above findings suggest that the mode of action of these novel iron halides is based on the induction of cellular damage by oxidative stress.

In addition, the study highlighted the differences in the most influential protein–protein interactions that were driving the response to iron-halide complexes. These observed findings revealed that the effect of the tetrabromide complex was mainly influenced by interactions among proteins involved in translation, ribosomal biogenesis and cell motility. However, the network analysis associated with the tetrachloride complex highlighted as a key driver the interaction of proteins involved in TCA cycle, oxidative phosphorylation, iron ion homeostasis and carbon/secondary metabolism. The identified molecular drivers suggested that iron complexes with dissimilar halide ligands could potentially lead to the generation of compounds with different capabilities. This dissimilarity in the proteomic profile of the bacteria exposed to different iron halides agrees with previous studies showing the association between antimicrobial activity and the nature of the halide ligand [99,102].

Hence, the findings revealed by this proteomic study suggest a common mode of action where a rise in free radicals caused by oxidative stress is next followed by the downregulation of key biological processes and cell damage. The dissimilarities in the proteomic changes induced by the iron-halide compounds may be associated with differences in the chemical properties of the halide ligands.

Importantly, the findings of this study can be used as a reference for comparing with the protein profile of a bacteria exposed to future variants of the iron-halide complexes including chemical modification and/or new delivery methods.

## Supplementary material

10.1099/acmi.0.000783.v4 Uncited Supplementary Material 1.

10.1099/acmi.0.000783.v4 Uncited Supplementary Material 2.

## Abbreviations

ANN: artificial neural network
GO: Gene Ontology
IDA: information-dependent acquisition
KEGG: Kyoto Encyclopedia of Genes and Genomes
LB: Luria–Bertani
ROS: reactive oxygen species
SAH: *S*-adenosylhomocysteine
SWATH: sequential window acquisition of all theoretical mass spectra
UPEC: uro-pathogenic *E. coli* strain
