# Supplementary material for: Protein profile of the Escherichia coli strain, BW25113, exposed to two novel iron-halide compounds: Fe(Hampy)2Cl4 and Fe(Hampy)2Br4
Source: Access Microbiol. 2025 Jan 28;7(1):000783.v4. doi: 10.1099/acmi.0.000783.v4 (PMC12282026; doi:10.1099/acmi.0.000783.v4)
Supplement: Uncited Supplementary Material 1. [file acmi-7-00783-s001.pdf]

Supplementary material for on-line submission

Table S1. Differentially expressed proteins of the *E. coli* exposed to the iron tetrachloride and iron tetrabromide compounds. The table is showing those deregulated proteins shared by both iron complexes. The values represent the results of the fold change of the deregulated proteins between treated vs untreated samples. Positive and negative values represent overexpression and downregulation respectively. Proteins with a positive fold change are highlighted with bold font. Selected proteins with a confidence value of ≥50 %.

		Fold change: Treated vs Control	
**Protein symbol**	**Protein name**	**Tetrachloride**	**Tetrabromide**
BamE	Outer membrane protein assembly factor BamE	-26.5786	-10.035
YraP	Uncharacterized protein YraP	-7.86179	-3.23993
YobBA	Protein YobA	-19.2336	-9.88325
RcsF	Outer membrane lipoprotein RcsF	-20.5971	-11.066
BtuB	Vitamin B12 transporter BtuB	-13.9318	-6.45491
YebY	Uncharacterized protein YebY	-14.3149	-3.51995
OsmB	Osmotically-inducible lipoprotein B	-56.4077	-14.0999
YbgS	Uncharacterized protein YbgS	-77.5473	-14.3061
RS21	30S ribosomal protein S21	-13.7242	-5.46984
EcnB	Entericidin B	-117.712	-22.2238
RL16	50S ribosomal protein L16	-11.0294	-15.9928
YifL	Uncharacterized lipoprotein YifL	-13.1387	-9.89209
RL19	50S ribosomal protein L19	-5.55804	-14.7749
YnhG	Probable L,D-transpeptidase YnhG	-20.59	-7.44088
FliC	Flagellin	-95.3857	-48.7144
YedD	Uncharacterized lipoprotein YedD	-11.2791	-4.75136
YehZ	Glycine betaine-binding protein YehZ	-5.78278	-4.04286
RS4	30S ribosomal protein S4	-11.378	-16.2743
RL5	50S ribosomal protein L5	-5.45524	-5.07559
MipA	MltA-interacting protein	-9.31864	-7.18684
RL2	50S ribosomal protein L2	-14.6673	-11.6886
RseA	Anti-sigma-E factor RseA	-11.1483	-9.98707
YciO	Uncharacterized protein YciO	-3.72571	-2.75908
McbA	Uncharacterized protein McbA	-35.8093	-12.5107
RS9	30S ribosomal protein S9	-19.4561	-22.4215
HdeA	Acid stress chaperone HdeA	-16.9482	-3.91705
YdeI	Uncharacterized protein YdeI	-60.8722	-16.4189
RS18	30S ribosomal protein S18	-6.43056	-7.22355
OsmY	Osmotically-inducible protein Y	-15.9734	-7.27215
LsrB	Autoinducer 2-binding protein LsrB	-6.26253	-5.15084
AstC	Succinylornithine transaminase	-22.0909	-14.065
SodC	Superoxide dismutase [Cu-Zn]	-27.2786	-10.8299
EftU	Elongation factor Tu 2	**3.411818**	-6.70346

LpoA	Penicillin-binding protein activator LpoA	-5.84595	-7.11948
YahO	Uncharacterized protein YahO	-38.309	-16.1839
YicS	Uncharacterized protein YicS	-18.0435	-9.36895
YhhA	Uncharacterized protein YhhA	-57.23	-10.3114
YgeR	Uncharacterized lipoprotein YgeR	-13.1623	-11.9926
G3p1	Glyceraldehyde-3-phosphate dehydrogenase A	**5.355786**	**3.33585**
OmpT	Protease 7	-23.2367	-13.5017
LamB	Maltoporin	-12.0629	-8.28106
AraF	L-arabinose-binding periplasmic protein	-10.0592	-6.29044
RS12	30S ribosomal protein S12	-18.8154	-12.3799
YccU	Uncharacterized protein YccU	-5.56289	-2.49597
YggN	Uncharacterized protein YggN	-15.1791	-3.69727
ClpB	Chaperone protein ClpB	-2.40252	-6.16513
YtfJ	Uncharacterized protein YtfJ	-6.9845	-6.20989
TsX	Nucleoside-specific channel-forming protein tsx	-10.9369	-10.7587
YbaY	Uncharacterized lipoprotein YbaY	-14.3338	-4.93119
FiU	Catecholate siderophore receptor Fiu	-28.6694	-10.2723
AG43	Antigen 43	-4.41825	-3.68162
YdgH	Protein YdgH	-6.05768	-5.01747
AcrB	Multidrug efflux pump subunit AcrB	-13.8505	**1.657265**
OmpX	Outer membrane protein X	-11.9841	-5.54443
RS19	30S ribosomal protein S19	-9.53777	-8.81701
FtsN	Cell division protein FtsN	-11.5243	-13.8669
PliG	Inhibitor of g-type lysozyme	-19.723	-9.69409
AhpC	Alkyl hydroperoxide reductase subunit C	**4.458448**	**3.814442**
Odp2	Dihydrolipoyllysine-residue acetyltransferase component of pyruvate dehydrogenase complex	**3.041148**	**2.591174**
DmsB	Anaerobic dimethyl sulfoxide reductase chain B	-7.03859	-15.5943
YebF	Protein YebF	-13.8954	-7.66754
PpiC	Peptidyl-prolyl cis-trans isomerase C	-11.7922	-7.85536
ArgT	Lysine/arginine/ornithine-binding periplasmic protein	-14.8567	-6.30327
CirA	Colicin I receptor	-17.0825	-10.2166
RL33	50S ribosomal protein L33	-9.31836	-13.1298
RL13	50S ribosomal protein L13	-5.61794	-10.028
TiG	Trigger factor	2.346563	-2.25157
RL22	50S ribosomal protein L22	-6.69632	-7.01444
RS3	30S ribosomal protein S3	-13.9108	-15.1164
YjjA	Uncharacterized protein YjjA	-11.0558	-2.54087
YqjC	Protein YqjC	-24.0945	-15.137
FecA	Fe(3+) dicitrate transport protein FecA	-12.6223	-10.4316
HdeB	Acid stress chaperone HdeB	-19.3717	-8.30778
YgfB	UPF0149 protein YgfB	**4.32089**	**3.198771**

MalZ	Maltodextrin glucosidase	**2.171686**	**2.994773**
RL15	50S ribosomal protein L15	-6.37366	-10.905
OsmE	Osmotically-inducible putative lipoprotein OsmE	-33.877	-16.2542
RS13	30S ribosomal protein S13	-11.4893	-13.2555
SodF	Superoxide dismutase [Fe]	**4.00382**	**8.918531**
SlP	Outer membrane protein slp	-4.78133	-5.13883
YnfD	Uncharacterized protein YnfD	-18.6047	-4.30338
MlaC	Probable phospholipid-binding protein MlaC	-17.793	-2.52602
YcfP	UPF0227 protein YcfP	-16.2442	-10.9145
TpX_	Thiol peroxidase	**2.066488**	**6.091261**
OdO2	Dihydrolipoyllysine-residue succinyltransferase component of 2-oxoglutarate dehydrogenase complex	**3.110733**	-2.16007
RS14	30S ribosomal protein S14	-13.9808	-12.2886
OmpW	Outer membrane protein W	-9.83421	-11.1808
FliY	L-cystine-binding protein FliY	-4.51794	**3.979391**
SlyB	Outer membrane lipoprotein SlyB	-11.6496	-6.46855
RlmN	Dual-specificity RNA methyltransferase RlmN	-8.59933	**5.748078**
ZnuA	High-affinity zinc uptake system protein ZnuA	-5.60412	-6.0666
OdP1	Pyruvate dehydrogenase E1 component	**2.355051**	**2.102921**
RL31	50S ribosomal protein L31	-6.53102	-16.0264
YbjP	Uncharacterized lipoprotein YbjP	-12.5776	-7.04132
GarR	2-hydroxy-3-oxopropionate reductase	-13.1681	-7.98411
RL4	50S ribosomal protein L4	-4.75708	-7.31209
YgdI	Uncharacterized lipoprotein YgdI	-10.6846	-9.55619
FadL	Long-chain fatty acid transport protein	-11.3837	-10.3968
BhsA	Multiple stress resistance protein BhsA	-10.2218	-8.88235
RS11	30S ribosomal protein S11	-9.50767	-11.1573
KpyK	Pyruvate kinase II	-5.46568	-7.76322
AlkH	KHG/KDPG aldolase	**3.396831**	**3.457486**
PsiF	Phosphate starvation-inducible protein PsiF	-48.6544	-32.6217
YnfB	UPF0482 protein YnfB	-7.83849	-3.39589
RL14	50S ribosomal protein L14	-8.54823	-7.34411
YoaB	RutC family protein YoaB	**6.082911**	**8.017129**
MlaA	Probable phospholipid-binding lipoprotein MlaA	-14.0568	-7.64324
YgiB	UPF0441 protein YgiB	-8.28381	-7.07762
RS5	30S ribosomal protein S5	-7.02509	-7.09732
GrcA	Autonomous glycyl radical cofactor	**6.716434**	**24.7588**
RL7	50S ribosomal protein L7/L12	**3.903064**	**2.77301**
RL27	50S ribosomal protein L27	-12.901	-7.14318
IVY	Inhibitor of vertebrate lysozyme	-4.89715	-3.12815
BsmA	Lipoprotein BsmA	-9.94687	-8.14136

RNI	Ribonuclease I	-11.0054	-11.3425
NfsB	Oxygen-insensitive NAD(P)H nitroreductase	**6.995164**	**7.139976**
YfcZ	UPF0381 protein YfcZ	**3.000842**	**10.36154**
YnjH	Uncharacterized protein YnjH	-29.0313	-6.66188
PurA	Adenylosuccinate synthetase	-3.23415	-4.29995
RS20	30S ribosomal protein S20	-11.7927	-4.64301
YtfQ	ABC transporter periplasmic-binding protein YtfQ	-7.81383	-5.57238
YdcS	Putative ABC transporter periplasmic-binding protein YdcS	-10.3505	-7.72935
AcP	Acyl carrier protein	**8.793442**	**7.972953**
YgiW	Protein YgiW	-9.37474	-2.91198
RoB	Right origin-binding protein	-2.66789	-6.92794
GabD	Succinate-semialdehyde dehydrogenase [NADP(+)] GabD	-3.40373	-7.24302
EftS	Elongation factor Ts	**2.335355**	**4.048965**
CpxP	Periplasmic protein CpxP	-6.81369	-5.70753
YidQ	Uncharacterized protein YidQ	-20.6998	-9.07731
YjeI	Uncharacterized protein YjeI	-32.2562	-10.6306
RS7	30S ribosomal protein S7	-11.1028	-8.41851
AcnA	Aconitate hydratase A	-3.84301	-4.30231
YdcA	Uncharacterized protein YdcA	-15.6371	-12.1587
RS8	30S ribosomal protein S8	-4.7967	-3.93011
TpiS	Triosephosphate isomerase	**2.204834**	**2.850668**
TalA	Transaldolase A	-3.63564	-3.66885
RL28	50S ribosomal protein L28	-21.9823	-10.4317
YqhD	Alcohol dehydrogenase YqhD	**4.0493**	**27.57479**
GlK	Glucokinase	-4.26058	-3.33944
YdcL	Uncharacterized lipoprotein YdcL	-9.12033	-4.63975
GlnB	Nitrogen regulatory protein P-II 1	**2.718312**	**2.341366**
PT1	Phosphoenolpyruvate-protein phosphotransferase	-2.21618	-1.64505
SpY	Periplasmic chaperone Spy	-9.23929	-3.61716
HinT	Purine nucleoside phosphoramidase	**3.110646**	**3.07802**
YmdF	Uncharacterized protein YmdF	-30.4407	-17.5664
GltI	Glutamate/aspartate import solute-binding protein	-3.59542	1.756256
FlgE	Flagellar hook protein FlgE	-55.0726	-4.68972
DpS	DNA protection during starvation protein	**4.479526**	-4.27521
RbsB	Ribose import binding protein RbsB	-3.30236	**3.227196**
RL6	50S ribosomal protein L6	-3.31015	-8.79988
MetK	S-adenosylmethionine synthase	**4.489602**	**5.40927**
RS2	30S ribosomal protein S2	-3.55018	-5.79213
Pur8	Adenylosuccinate lyase	-8.4497	-5.16499
KbL	2-amino-3-ketobutyrate coenzyme A ligase	-3.1504	-3.76266
NfuA	Fe/S biogenesis protein NfuA	**4.298638**	**8.144253**

SecD	Protein translocase subunit SecD	-54.3578	-20.0168
PspA	Phage shock protein A	**3.640836**	**3.85181**
CspA	Cold shock protein CspA	**5.444408**	24.36782
SyT	Threonine--tRNA ligase	**3.239909**	-6.49251
CreA	Protein CreA	-10.7575	-5.89211
RidA	2-iminobutanoate/2-iminopropanoate deaminase	**5.236437**	**4.951452**
ErfK	Probable L,D-transpeptidase ErfK/SrfK	-23.2151	-5.19545
NqoR	NAD(P)H dehydrogenase (quinone)	**1.751297**	-3.35543
YncE	Uncharacterized protein YncE	-10.5251	-9.83943
HchA	Protein deglycase 1	-4.18953	-4.94523
FhuA	Ferrichrome-iron receptor	-14.0546	-11.7306
AgaL	Alpha-galactosidase	2.413141	-7.3454
HpF	Ribosome hibernation promoting factor	-3.41646	-3.87892
PtgA	PTS system glucose-specific EIIA component	**2.112454**	**3.237975**
RL17	50S ribosomal protein L17	-5.15902	-7.70779
YjbJ	UPF0337 protein YjbJ	-6.56691	-4.79597
HnS	DNA-binding protein H-NS	**4.027613**	-3.56993
AspA	Aspartate ammonia-lyase	**2.19272**	-3.99665
YedF	Putative sulfur carrier protein YedF	**2.817005**	**2.341584**
YbhB	UPF0098 protein YbhB	-3.34097	**2.500852**
GlrX3	Glutaredoxin 3	-5.01419	-1.86104
GpmA	2,3-bisphosphoglycerate-dependent phosphoglycerate mutase	-3.24747	-2.42278
AlF1	Fructose-bisphosphate aldolase class 1	**3.190615**	-4.80076
AckA	Acetate kinase	-2.7401	-4.21992
CisY	Citrate synthase	**3.185819**	-6.09963
6PgD	6-phosphogluconate dehydrogenase, decarboxylating	**1.795297**	-1.68041
ThiO	Thioredoxin 1	-2.78379	**6.283399**
MalM	Maltose operon periplasmic protein	-3.73017	-6.6583
NuoH	NADH-quinone oxidoreductase subunit H	-4.89117	-4.91362
RL3	50S ribosomal protein L3	-2.87126	-6.14893
RpoA	DNA-directed RNA polymerase subunit alpha	**2.612913**	-3.42934
YgaU	Uncharacterized protein YgaU	-6.88513	-6.59245
NlpD	Murein hydrolase activator NlpD	-4.74822	-3.60071
YmgD	Uncharacterized protein YmgD	-2.88637	**1.944666**
TkT2	Transketolase 2	-2.23789	-8.24869
FlAV	Flavodoxin 1	**4.346121**	**6.384835**
YeaY	Uncharacterized lipoprotein YeaY	-7.62531	-6.00611
SrA	Stationary-phase-induced ribosome-associated protein	-3.02734	-11.3897
FepA	Ferrienterobactin receptor	-16.6024	-9.67245
EntF	Enterobactin synthase component F	-17.6455	-1.72309
RL32	50S ribosomal protein L32	-6.45103	-8.68493

DhaL	PEP-dependent dihydroxyacetone kinase, ADP-binding subunit DhaL	**3.091**	**6.291842**
LptE	LPS-assembly lipoprotein LptE	-8.9883	-5.02061
SyS	Serine--tRNA ligase	**2.255968**	-4.06372
FabI	Enoyl-[acyl-carrier-protein] reductase [NADH] FabI	-5.35011	-2.85193
CH60	60 kDa chaperonin	-2.19475	-3.17955
RimP	Ribosome maturation factor RimP	**4.693531**	**5.242039**
YbaV	Uncharacterized protein YbaV	-6.47034	-7.65456
YaeH	UPF0325 protein YaeH	-2.08468	-5.04547
RL10	50S ribosomal protein L10	**2.80166**	-3.96023
PflB	Formate acetyltransferase 1	-1.96584	-4.15429
RS10	30S ribosomal protein S10	-2.135	-3.41795
Map1	Methionine aminopeptidase	**4.436361**	**5.579595**
IscU	Iron-sulfur cluster assembly scaffold protein IscU	**5.032679**	**11.97709**
AdhE	Aldehyde-alcohol dehydrogenase	-2.68019	-5.73146
FabA	3-hydroxydecanoyl-[acyl-carrier-protein] dehydratase	**4.300519**	**3.673839**
DnaK	Chaperone protein DnaK	-2.00383	-1.42963
EcoT	Ecotin	-5.3621	**2.260346**
YbiJ	Uncharacterized protein YbiJ	-47.482	-9.38275
AceA	Isocitrate lyase	**3.061789**	-8.90354
RL9	50S ribosomal protein L9	-2.29854	-3.15
CrP	cAMP-activated global transcriptional regulator CRP	-3.75556	-6.06638
GlgS	Surface composition regulator	-13.3036	**1.60669**
RL24	50S ribosomal protein L24	-4.00155	-10.1966
PthP	Phosphocarrier protein HPr	-4.25991	**4.159735**
RrF	Ribosome-recycling factor	**1.830223**	**4.429878**
YcdX	Probable phosphatase YcdX	**6.319877**	**6.887438**
TnaA	Tryptophanase	**7.625093**	-9.00217
YdcH	Uncharacterized protein YdcH	-6.26491	-9.51456
YdfZ	Putative selenoprotein YdfZ	-2.60245	**3.687698**
OsmC	Peroxiredoxin OsmC	**7.795839**	**2.724231**
PanD	Aspartate 1-decarboxylase	**2.856394**	**9.82005**
MscM	Miniconductance mechanosensitive channel MscM	-13.4622	-2.23815
AzoR	FMN-dependent NADH-azoreductase	**5.789583**	**9.490978**
ApT	Adenine phosphoribosyltransferase	**4.358528**	**4.377133**
YegQ	Uncharacterized protease YegQ	**3.85311**	**7.681257**
RmlB1	dTDP-glucose 4,6-dehydratase 1	-3.22271	-9.12818
YbaB	Nucleoid-associated protein YbaB	**9.586321**	**10.42048**
AldB	Aldehyde dehydrogenase B	-2.92271	-4.3708
YejL	UPF0352 protein YejL	**4.090137**	**9.253615**
LuxS	S-ribosylhomocysteine lyase	**2.479649**	**4.097505**
RS6	30S ribosomal protein S6	**1.916942**	-5.11285
EfpL	Elongation factor P-like protein	**6.567902**	**2.922779**

GpmI	2,3-bisphosphoglycerate-independent phosphoglycerate mutase	**2.006881**	**3.390448**
YcaR	UPF0434 protein YcaR	-2.56991	3.084028
PncA	Nicotinamidase	**2.483646**	**7.460105**
DkgA	2,5-diketo-D-gluconic acid reductase A	-2.48482	**5.107037**
CysP	Thiosulfate-binding protein	-1.98285	**4.521474**
ErpA	Iron-sulfur cluster insertion protein ErpA	**7.221165**	**13.32839**

Table S2. Differentially expressed proteins of the *E. coli* exposed to the iron tetrabromide compound. The table is showing the expression of the proteins specifically deregulated by the tetrabromide complex. The values represent the results of the fold change of the deregulated proteins between treated vs untreated samples. Positive and negative values represent overexpression and downregulation respectively. Proteins with a positive fold change are highlighted with bold font. Selected proteins with a confidence value of ≥50 %.

Protein	Protein name	Fold change: treated vs Control
SufA	Protein SufA	**8.852972792**
TolB	Protein TolB	**4.613338165**
YtfP	Gamma-glutamylcyclotransferase family protein YtfP	**3.409476803**
PstS	Phosphate-binding protein PstS	**7.980156054**
KcY	Cytidylate kinase	**4.031506622**
PtsN	Nitrogen regulatory protein	**8.192043712**
FeR	2Fe-2S ferredoxin	**14.4291825**
GreA	Transcription elongation factor GreA	**3.034548576**
HspQ	Heat shock protein HspQ	**10.66317071**
CspE	Cold shock-like protein CspE	**6.60230595**
BamA	Outer membrane protein assembly factor BamA	**4.562813862**
SucC	Succinate--CoA ligase [ADP-forming] subunit beta	-6.94218855
MsrC	Free methionine-R-sulfoxide reductase	**7.860174115**
KatG	Catalase-peroxidase	-4.454711865
RS1	30S ribosomal protein S1	-3.472298425
CoaB	Coenzyme A biosynthesis bifunctional protein CoaBC	**9.41671007**
YecJ	Uncharacterized protein YecJ	**6.065061107**
KpyK1	Pyruvate kinase I	-4.438672509
RpoB	DNA-directed RNA polymerase subunit beta	-5.592675957
FlaW	Flavodoxin 2	**4.214140887**
RpoC	DNA-directed RNA polymerase subunit beta'	-6.250094045
YccJ	Uncharacterized protein YccJ	**3.275903305**
EfG	Elongation factor G	-5.325790601
CspC	Cold shock-like protein CspC	**5.218991631**
RcnB	Nickel/cobalt homeostasis protein RcnB	**6.569998566**

RpoZ	DNA-directed RNA polymerase subunit omega	**5.908476918**
YjiM	Uncharacterized protein YjiM	**7.096853694**
YgaM	Uncharacterized protein YgaM	-7.695345953
BfR	Bacterioferritin	-3.747086883
YhjJ	Protein YhjJ	**6.817311894**
RL1	50S ribosomal protein L1	-3.949057587
DegP	Periplasmic serine endoprotease DegP	**2.709941174**
IscA	Iron-binding protein IscA	**11.85539035**
IF1	Translation initiation factor IF-1	**10.22145843**
DeF	Peptide deformylase	**5.748693954**
YceD	Large ribosomal RNA subunit accumulation protein YceD	**7.208297121**
YceF	Maf-like protein YceF	-5.538383741
SubI	Sulfate-binding protein	**9.556995191**
PoxB	Pyruvate dehydrogenase [ubiquinone]	-4.741360885
AldA	Lactaldehyde dehydrogenase	-6.752718884
YajQ	UPF0234 protein YajQ	**1.780005349**
EnO	Enolase	-4.116257309
ToP1	DNA topoisomerase 1	-4.374320273
DsbA	Thiol:disulfide interchange protein DsbA	**2.802042847**
YhhW	Quercetin 2,3-dioxygenase	**8.057344429**
RL25	50S ribosomal protein L25	-5.425523091
YgiIN	Probable quinol monooxygenase YgiN	**6.668974198**
DeoC	Deoxyribose-phosphate aldolase	**2.525690676**
SyK1	Lysine--tRNA ligase	-5.74131495
DsbC	Thiol:disulfide interchange protein DsbC	**3.836609053**
HldD	ADP-L-glycero-D-manno-heptose-6-epimerase	-7.080214881
AccD	Acetyl-coenzyme A carboxylase carboxyl transferase subunit beta	-4.101176747
PotD	Spermidine/putrescine-binding periplasmic protein	**2.046539108**
SyfB	Phenylalanine--tRNA ligase beta subunit	-3.958065498
AcuI	Probable acrylyl-CoA reductase AcuI	**2.330815685**
RoF	Protein rof	**6.812977869**
HtpG	Chaperone protein HtpG	-2.744004553
EnD4	Endonuclease 4	**6.084253722**
NadR	Trifunctional NAD biosynthesis/regulator protein NadR	**4.467715903**
UdP	Uridine phosphorylase	-2.471633806
TalB	Transaldolase B	**1.84481034**

Table S3. Differentially expressed proteins of the *E. coli* exposed to the iron tetrachloride compound. The table is showing the expression of the proteins specifically deregulated by the tetra-chloride complex. The values represent the results of the fold change of the deregulated proteins between treated vs untreated samples. Positive and negative values represent overexpression and downregulation respectively. Proteins with a positive fold change are highlighted with bold font. Selected proteins with a confidence value of ≥50 %.

Protein	Protein name	Fold change: Treated vs Control
CysK	Cysteine synthase A	**4.705581**
YdiZ	Uncharacterized protein YdiZ	-6.03334
SdhA	Succinate dehydrogenase flavoprotein subunit	**7.14011**
AtpA	ATP synthase subunit alpha	**3.165266**
TkT1	Transketolase 1	**2.979615**
ThiS	Sulfur carrier protein ThiS	**4.64164**
SdhB	Succinate dehydrogenase iron-sulfur subunit	**5.912212**
YoaF	Uncharacterized protein YoaF	-15.5185
CspD	Cold shock-like protein CspD	**8.049539**
OmpC	Outer membrane protein C	-8.95632
ElaB	Protein ElaB	**3.571189**
ManA	Mannose-6-phosphate isomerase	**6.155188**
SyE	Glutamate--tRNA ligase	**3.487249**
SyN	Asparagine--tRNA ligase	-2.14657
SyP	Proline--tRNA ligase	**5.203908**
FtnA	Bacterial non-heme ferritin	**3.635968**
YajG	Uncharacterized lipoprotein YajG	-7.99294
FrdB	Fumarate reductase iron-sulfur subunit	**6.912644**
AtpL	ATP synthase subunit c	**11.67795**
BasR	Transcriptional regulatory protein BasR	**8.015808**
DldH	Dihydrolipoyl dehydrogenase	**3.785534**
PckA	Phosphoenolpyruvate carboxykinase (ATP)	**2.496218**
PtkC	PTS system galactitol-specific EIIC component	**6.654204**
FabF	3-oxoacyl-[acyl-carrier-protein] synthase 2	**2.910484**
PepB	Peptidase B	**5.327992**
GatD	Galactitol 1-phosphate 5-dehydrogenase	**5.755094**
DhpS	Dihydropteroate synthase	**7.240222**
YihD	Protein YihD	**2.600222**
ArtI	Putative ABC transporter arginine-binding protein 2	-7.68739
FtsH	ATP-dependent zinc metalloprotease FtsH	-9.41723
HisJ	Histidine-binding periplasmic protein	-5.27811
ThiC	Phosphomethylpyrimidine synthase	**6.094854**
SyA	Alanine--tRNA ligase	**4.365157**
TolC	Outer membrane protein TolC	-2.19768
SyY	Tyrosine--tRNA ligase	**2.440007**

IlvN	Acetolactate synthase isozyme 1 small subunit	**5.322698**
ArnB	UDP-4-amino-4-deoxy-L-arabinose--oxoglutarate aminotransferase	**9.862344**
MdH	Malate dehydrogenase	**1.948745**
SyV	Valine--tRNA ligase	**2.798051**
DcP	Dipeptidyl carboxypeptidase	**3.491525**
YobB	Uncharacterized protein YobB	-14.0347
YeaG	Uncharacterized protein YeaG	-15.6326
HslU	ATP-dependent protease ATPase subunit HslU	-8.403
MasY	Malate synthase A	**2.621459**
CaN	Carbonic anhydrase 2	**5.535199**
EfeO	Iron uptake system component EfeO	-5.8052
PpA	Periplasmic AppA protein	-7.05742
MoaB	Molybdenum cofactor biosynthesis protein B	**5.09397**
TreA	Periplasmic trehalase	-14.8628
AtpD	ATP synthase subunit delta	**4.02426**
KaD	Adenylate kinase	**4.014909**
LPTA	Lipopolysaccharide export system protein LptA	-8.84348
TesA	Acyl-CoA thioesterase 1	-6.6644
ImdH	Inosine-5'-monophosphate dehydrogenase	**2.130146**
PtrB	Protease 2	-9.56755
PpiA	Peptidyl-prolyl cis-trans isomerase A	-5.31378
SpeB	Agmatinase	**3.796858**
GlnH	Glutamine-binding periplasmic protein	-7.03558
SyD	Aspartate--tRNA ligase	**3.095752**
SygB	Glycine--tRNA ligase beta subunit	-4.48766
DmlA	D-malate dehydrogenase [decarboxylating]	**5.570925**
ModA	Molybdate-binding periplasmic protein	-6.83653
G6pI	Glucose-6-phosphate isomerase	**2.280469**
RisB	6,7-dimethyl-8-ribityllumazine synthase	**5.603609**
LoN	Lon protease	-10.9488
YchN	Protein YchN	**3.944404**
PanB	3-methyl-2-oxobutanoate hydroxymethyltransferase	**4.042481**
MasZ	Malate synthase G	**2.688219**
QseB	Transcriptional regulatory protein QseB	**5.909114**
YibT	Uncharacterized protein YibT	-7.12861
YodD	Uncharacterized protein YodD	-5.17189
Mao2	NADP-dependent malic enzyme	**2.90009**
ThiG	Thiazole synthase	**8.160466**
CopA	Copper-exporting P-type ATPase A	-5.4696
AdA	Bifunctional transcriptional activator/DNA repair enzyme Ada	-3.78888
FkbB	FKBP-type 22 kDa peptidyl-prolyl cis-trans isomerase	**3.051583**

IadA	Isoaspartyl dipeptidase	**8.196738**
BlC	Outer membrane lipoprotein Blc	-10.4869
BioH	Pimeloyl-[acyl-carrier protein] methyl ester esterase	**15.79012**
AaT	Aspartate aminotransferase	**2.262849**
YdcF	Protein YdcF	-4.64675
YajD	Uncharacterized protein YajD	**14.07984**
OppA	Periplasmic oligopeptide-binding protein	-15.7438
OmpA	Outer membrane protein A	-6.83267
KdsA	2-dehydro-3-deoxyphosphooctonate aldolase	-4.60025
IpyR	Inorganic pyrophosphatase	**3.860854**
SpeD	S-adenosylmethionine decarboxylase proenzyme	**6.462016**
TusB	Protein TusB	**8.605243**
RL18	50S ribosomal protein L18	**3.440396**
SyfA	Phenylalanine--tRNA ligase alpha subunit	**3.034911**
YaeP	UPF0253 protein YaeP	-6.57478
MoaE	Molybdopterin synthase catalytic subunit	**4.174432**
YajC	UPF0092 membrane protein YajC	**4.55504**
DbhA	DNA-binding protein HU-alpha	**3.507345**
HeM2	Delta-aminolevulinic acid dehydratase	**2.833687**
DapD	2,3,4,5-tetrahydropyridine-2,6-dicarboxylate N-succinyltransferase	**2.451911**
FabG	3-oxoacyl-[acyl-carrier-protein] reductase FabG	-2.12198
YgjR	Uncharacterized oxidoreductase YgjR	-10.4669
PanE	2-dehydropantoate 2-reductase	**3.283826**
ClpP	ATP-dependent Clp protease proteolytic subunit	**2.261759**
YjdI	Uncharacterized protein YjdI	-9.99633
AK3	Lysine-sensitive aspartokinase 3	**7.798005**

Table S4. Cluster composition of the interactome associated to the iron tetrabromide. The table enlists the hubs identified along with the proteins that form part of the FLIC and the ECNB/MVBA networks. The values represent the results of the fold change of the deregulated proteins between treated vs untreated samples. Negative values represent downregulation. Selected proteins with a confidence value of ≥50 %.

	**FLIC cluster**	
**Protein symbol**	**Protein name**	**Fold change: Treated vs Control**
FliC	Flagellin	-48.7144
RS2	30S ribosomal protein S2	-5.79213
RS21	30S ribosomal protein S21	-5.46984
RS20	30S ribosomal protein S20	-4.64301

RL5	50S ribosomal protein L5	-5.07559
RS13	30S ribosomal protein S13	-13.2555
RS9	30S ribosomal protein S9	-22.4215
YedD	Uncharacterized lipoprotein YedD	-4.75136
RS12	30S ribosomal protein S12	-12.3799
RL14	50S ribosomal protein L14	-7.34411
RL4	50S ribosomal protein L4	-7.31209
RL15	50S ribosomal protein L15	-10.905
RL16	50S ribosomal protein L16	-15.9928
RL22	50S ribosomal protein L22	-7.01444
RL13	50S ribosomal protein L13	-10.028
RL28	50S ribosomal protein L28	-10.4317
RS18	30S ribosomal protein S18	-7.22355
RS19	30S ribosomal protein S19	-8.81701
RL6	50S ribosomal protein L6	-8.79988
YifL	Uncharacterized lipoprotein YifL	-9.89209
YggN	Uncharacterized protein YggN	-3.69727
FecA	Fe(3+) dicitrate transport protein FecA	-10.4316
RcsF	Outer membrane lipoprotein RcsF	-11.066
YhhA	Uncharacterized protein YhhA	-10.3114
AckA	Acetate kinase	-4.21992
RL32	50S ribosomal protein L32	-8.68493
RS4	30S ribosomal protein S4	-16.2743
RS3	30S ribosomal protein S3	-15.1164
RNI	Ribonuclease I	-11.3425
McbA	Uncharacterized protein McbA	-12.5107
	**ECNB/MVBA cluster**	
EcnB	Entericidin B	-22.2238
MvbA	Uncharacterized protein McbA	-12.5107

Table S5. Cluster composition of the interactome associated to the bacteria strain exposed to iron tetrachloride. The table enlists the hubs identified along with the proteins that form part of the METK and the YDEI/OSMY networks. The values represent the results of the fold change of the deregulated proteins between treated vs untreated samples. Positive and negative values represent overexpression and downregulation respectively. Proteins with a positive fold change are highlighted with bold font. Selected proteins with a confidence value of ≥50 %.

	METK cluster	
**Protein symbol**	**Protein name**	**Fold change: Treated vs Control**
MetK	S-adenosylmethionine synthase	**4.489602**
MoaB	Molybdenum cofactor biosynthesis protein B	**5.09397**
AgaL	Alpha-galactosidase	**2.413141**
SdhB	Succinate dehydrogenase iron-sulfur subunit	**5.912212**
SyY	Tyrosine--tRNA ligase	**2.440007**

DldH	Dihydrolipoyl dehydrogenase	**3.785534**
PtkC	PTS system galactitol-specific EIIC component	**6.654204**
OdP2	Dihydrolipoyllysine-residue acetyltransferase component of pyruvate dehydrogenase complex	**3.041148**
AlF1	Fructose-bisphosphate aldolase class 1	**3.190615**
SyV	Valine--tRNA ligase	**2.798051**
DpS	DNA protection during starvation protein	**4.479526**
AspA	Aspartate ammonia-lyase	**2.19272**
SyT	Threonine--tRNA ligase	**3.239909**
ThiG	Thiazole synthase	**8.160466**
FrdB	Fumarate reductase iron-sulfur subunit	**6.912644**
RL7	50S ribosomal protein L7/L12	**3.903064**
PepB	Peptidase B	**5.327992**
FtnA	Bacterial non-heme ferritin	**3.635968**
OdP1	Pyruvate dehydrogenase E1 component	**2.35505**1
SyD	Aspartate--tRNA ligase	**3.095752**
MaO2	NADP-dependent malic enzyme	**2.90009**
DmlA	D-malate dehydrogenase [decarboxylating]	**5.570925**
AtpL	ATP synthase subunit c	**11.67795**
GlnB	Nitrogen regulatory protein P-II 1	**2.718312**
OdO2	Dihydrolipoyllysine-residue succinyltransferase component of 2-oxoglutarate dehydrogenase complex	**3.110733**
	**YDEI/OSMY cluster**	
YdeI	Uncharacterized protein YdeI	-60.8722
OsmY	Osmotically-inducible protein Y	-15.9734
AstC	Succinylornithine transaminase	-22.0909
SpY	Periplasmic chaperone Spy	-9.23929
YbhB	UPF0098 protein YbhB	-3.34097
YmdF	Uncharacterized protein YmdF	-30.4407

Table S6. Cluster composition of the interactome associated to the iron tetrabromide. This analysis was based on the significantly deregulated proteins that were shared between the $Fe(HL)_2Cl_4$ and $Fe(HL)_2Br_4$ complexes. The table enlists the hubs identified along with the proteins that form part of the CLPB/SLP cluster. The values represent the results of the fold change of the deregulated proteins between treated vs untreated samples. Negative values represent downregulation. Selected proteins with a confidence value of ≥50 %.

	**CLPB/SLP cluster**	
**Protein symbol**	**Protein name**	**Fold change: Treated vs Control**
YqjC	Protein YqjC	-15.137
RS19	30S ribosomal protein S19	-8.81701
FiU	Catecholate siderophore receptor Fiu	-10.2723

RS8	30S ribosomal protein S8	-3.93011
RS13	30S ribosomal protein S13	-13.2555
YdcA	Uncharacterized protein YdcA	-12.1587
YCI0	Uncharacterized protein YciO	-2.75908
RL6	50S ribosomal protein L6	-8.79988
RS3	30S ribosomal protein S3	-15.1164
RL19	50S ribosomal protein L19	-14.7749
RL22	50S ribosomal protein L22	-7.01444
RS7	30S ribosomal protein S7	-8.41851
SecD	Protein translocase subunit SecD	-20.0168
RS4	30S ribosomal protein S4	-16.2743
RL16	50S ribosomal protein L16	-15.9928
RL13	50S ribosomal protein L13	-10.028
RnI	Ribonuclease I	-11.3425
RscF	Outer membrane lipoprotein RcsF	-11.066
FtsN	Cell division protein FtsN	-13.8669
FecA	Fe(3+) dicitrate transport protein FecA	-10.4316
YedD	Uncharacterized lipoprotein YedD	-4.75136
RS21	30S ribosomal protein S21	-5.46984
RS12	30S ribosomal protein S12	-12.3799
RS18	30S ribosomal protein S18	-7.22355
KpyK2	Pyruvate kinase II	-7.76322
FliC	Flagellin	-48.7144
YggN	Uncharacterized protein YggN	-3.69727
RL28	50S ribosomal protein L28	-10.4317
RL19	50S ribosomal protein L19	-14.7749
RL4	50S ribosomal protein L4	-7.31209
RS2	30S ribosomal protein S2	-5.79213
RL14	50S ribosomal protein L14	-7.34411
RL5	50S ribosomal protein L5	-5.07559
RS20	30S ribosomal protein S20	-4.64301
RS9	30S ribosomal protein S9	-22.4215

Table S7. Cluster composition of the interactome associated to the iron tetrachloride. This analysis was based on the significantly deregulated proteins that were shared between the tetrachloride and tetrabromide complexes. The table enlists the hubs identified along with the proteins that form part of the YBG5 cluster. The values represent the results of the fold change of the deregulated proteins between treated vs untreated samples. Positive and negative values represent overexpression and downregulation respectively. Proteins with a positive fold change are highlighted with bold font. Selected proteins with a confidence value of ≥50 %.

	**YBG5 cluster**	
**Protein symbol**	**Protein name**	**Fold Change: Treated vs Control**
McbA	Uncharacterized protein McbA	-35.8093
SpY	Periplasmic chaperone Spy	-9.23929
GpmA	2,3-bisphosphoglycerate-dependent phosphoglycerate mutase	-3.24747
ArgT	Lysine/arginine/ornithine-binding periplasmic protein	-14.8567
AstC	Succinylornithine transaminase	-22.0909
PsiF	Phosphate starvation-inducible protein PsiF	-48.6544
YbhB	UPF0098 protein YbhB	-3.34097
YebF	Protein YebF	-13.8954
YdeI	Uncharacterized protein YdeI	-60.8722
EcnB	Entericidin B	-117.712
HchA	Protein deglycase 1	-4.18953
YbaY	Uncharacterized lipoprotein YbaY	-14.3338
YebF	Protein YebF	-13.8954
DmsB	Anaerobic dimethyl sulfoxide reductase chain B	-7.03859
YfcZ	UPF0381 protein YfcZ	**3.000842**
CspA	Cold shock protein CspA	**5.444408**
TalLA	Transaldolase A	-3.63564
YgdI	Uncharacterized lipoprotein YgdI	-10.6846
YdcL	Uncharacterized lipoprotein YdcL	-9.12033
